# Prevalence of Fibromyalgia Syndrome in Taif City, Saudi Arabia

**DOI:** 10.7759/cureus.32489

**Published:** 2022-12-13

**Authors:** Nawaf K Althobaiti, Bashir A Amin, Abdulrahman D Alhamyani, Sultan M Alzahrani, Abdulrahman M Alamri, Faisal Khaled H Alhomayani

**Affiliations:** 1 Medicine, Taif University, Taif, SAU; 2 Nephrology, Taif University, Taif, SAU

**Keywords:** taif city, awareness, prevalence, fms, fibromyalgia syndrome, fibromyalgia

## Abstract

Introduction

Fibromyalgia syndrome (FMS) is a non-inflammatory, chronic disseminated musculoskeletal pain with unknown etiology. FMS patients suffer from generalized pain that markedly decreases their quality of life and productivity.

Objective

To investigate the prevalence of FMS and the correlation between people with positive screening criteria for FMS and their socio-demographic characteristics in Taif city.

Methodology

A cross-sectional study was performed in Taif city, Saudi Arabia, from June 2021 to August 2021. A structured self-estimated electronic questionnaire developed by Google Forms. The questionnaire depended on the 2010 American College of Rheumatology (ACR) criteria. Data analysis was performed by using SPSS, version 21.0 (IBM Corp., Armonk, NY).

Result

Out of 1015 participants, 77 participants (7.6%) were revealed to have FMS. The prevalence of FMS among females (9.3%) was significantly higher (p<0.001) than that among males (3.1%). In addition, participants aged 40 years old or more showed a significantly higher prevalence of FMS (p=0.003) compared to those aged less than 40 years old (11.7% versus 6.0%, respectively). In addition, occupational status was found to significantly affect FMS prevalence (p=0.040) as the highest prevalence was reported among employees (10.8%) and housewives (9.4%) compared to the unemployed (8.8%), students (5.0%), and retired participants (4.0%). On the other hand, participants’ nationality was shown to have no significant effect on fibromyalgia prevalence (p=0.396).

Conclusion

Results show a slightly high prevalence rate of FMS in Saudi Arabia. Prevalence was seen greater in women, old age, and employed individuals. Poor knowledge of FMS was seen among the general Saudi population. Educational programs are needed to increase awareness of the disease.

## Introduction

Fibromyalgia syndrome (FMS) is a non-inflammatory, chronic disseminated musculoskeletal pain that has an environmental and genetic factor with unknown etiology [1-3]. FMS is considered a common issue in rheumatology practice in different countries [4]. Patients with FMS suffer from generalized pain which is described as a deep, throbbing, persistent, and intense pain in muscles that markedly decrease the quality of life and productivity [5,6]. Also, FMS patients might have tenderness, sleep disorder, fatigue, headache, and psychological disorders alongside the pain but the clinical picture could fluctuate extensively in the same patient [7,8]. However, the disabling pain will not cause tissue damage [9]. Regarding the risk factors, psychological stress and sleep deprivation play a significant role in pathophysiology [3,10]. Women are at high risk for FMS with rates seven to nine times than men [11]. Also, a family history of FMS, rheumatological conditions, and age between 20 and 60 years are other important risk factors [12].

The previous diagnostic criteria for FMS established in 1990 by the American College of Rheumatology (ACR) was mainly dependent on tender points examination. while the new 2010 ACR criteria are based on two components; which are severity score (SS) and widespread pain index (WPI) with abolishing of examination of tender points [13-15]. In addition, multiple tests may be ordered to investigate other possible differential diagnoses of patients’ symptoms, such as systemic lupus erythematosus and rheumatoid arthritis. Although the exact etiology of fibromyalgia is still unclear, several factors including dysfunction of the CNS, autonomic nervous system, hormones, immune system, and neurotransmitters, seem to be involved. In addition, external stressors and psychological status can cause fibromyalgia [16]. FMS is a common issue worldwide in all socio-economic and ethnic groups [17,18]. Since the awareness of FMS and its accompanied socioeconomic burden, the epidemiological studies on the population have increased. Still, any study on the national level to assess the magnitude of the prevalence of the disease is lacking [3]. Only two studies were conducted on special populations in Riyadh and Jeddah cities [1,3]. So, in view of the impact of FMS on the health care system and the unavailability of any study in Taif city evaluating the epidemiology of FMS, we decided to conduct a cross-sectional study among Taif city residents and find out the correlation between individuals with positive screening criteria of FMS and their socio-demographic characteristics.

## Materials and methods

A cross-sectional study was performed at Taif city, Makkah region, Kingdom of Saudi Arabia (KSA), from June 2021 to August 2021. The institutional research ethics board approval was obtained from Taif University before performing any study procedure (approval No. 43-013). The identities of the participants are kept confidential.

A structured self-estimated electronic questionnaire developed by Google Forms was distributed to Taif city residents aged 20 years and above. Each responder was given informed consent before filling out the survey. The study included all individuals who consented to participate. Individuals were invited to finish the Fibromyalgia Survey Diagnostic Criteria (FSDC) validated in a prior cross-sectional study that included 1,651 FMS patients as it depended on the 2010 diagnostic criteria for FMS of the ACR [19]. Also, the study included individuals who were previously diagnosed with FMS. All individuals aged less than 20 years were excluded.

The minimum sample size required for the confidence level of 95% (CI-95%) based on the calculation equation was computed to be 384 [20]. However, the number of individuals who participated in this study was 1015. The selection technique of participants was a simple random sampling method.

The questionnaire of the study involved socio-demographic characteristics, including age, sex, nationality, body mass index (BMI), monthly income, marital status, educational level, and occupation. Furthermore, responders were further asked about smoking, the history of chronic comorbidities, and the number of hours that the participants slept per night for the past month. In addition to that, they queried whether they were taking any pain-relieving medications or if their exercises were affected by the pain. Moreover, they were questioned if they have suffered from emotional stress from their family or friends due to their chronic pain.

FSDC contains the Symptom Severity Score (SSS) that reports the three main somatic symptoms of FMS: waking unrefreshed, cognitive symptoms, and fatigue over the past seven days that addressed (0-12 as the final score), and WPI, which show the number (a 0-19 count) of painful areas that described by the participant in the past seven days.

The identification of participants with positive results for FMS was done by using the ACR 2010 diagnostic criteria for FMS, which involves SSS and WPI [21]. The positive response is considered whether the response was meeting a ≥7/19 of WPI score for the pain site and a ≥5/12 of SSS score or if the responders achieved a number between 3-6/19 of WPI score and a ≥9/12 of SSS score. Statistical analysis was done by using SPSS, version 21.0 (IBM Corp., Armonk, NY). Descriptive statistics were performed to show the rates of responses, socio-demographic characteristics, and FMS prevalence.

## Results

A total of 1015 individuals participated in the current study. More than half of the participants (52.6%) were aged between 20 and 29 years and the vast majority (88.7%) were aged between 20 and 49 years. The largest proportions of the participants were Saudi (95.5%) females (71.8%), single (50%), students (43.1%), and held University or higher degrees (78.6%). More details are provided in Table 1.

**Table 1:** Participants’ characteristics n=327 (261 females and 66 males) is the number of participants who feel pain in several parts of their bodies for at least three months that is not resulting from an accident or injury. FMS: Fibromyalgia Syndrome

	Male n= 286 Count (%)	Female n= 729 Count (%)	Total n= 1015 Count (%)
Age			
20 – 29 years	164 (57.3%)	370 (50.8%)	534 (52.6%)
30 – 39 years	39 (13.6%)	159 (21.8%)	198 (19.5%)
40- 49 years	30 (10.5%)	138 (18.9%)	168 (16.6%)
50 – 59 years	35(12.2%)	56 (7.7%)	91 (9.0%)
60 – 69 years	16 (5.6%)	6 (0.8%)	22 (2.2%)
70 – 79 years	2 (0.7%)	0 (0%)	2 (0.2%)
Marital status			
Single	167 (58.4%)	341 (46.8%)	508 (50%)
Married	117 (40.9%)	354 (48.6%)	471 (46.4%)
Divorced	0 (0%)	24 (3.3%)	24 (2.4%)
Widowed	2 (0.7%)	10 (1.4%)	12 (1.2%)
Occupation			
Employee	102 (35.7%)	176 (24.1%)	278 (27.4%)
Housewife	0 (0%)	169 (23.2%)	169 (16.7%)
Retired	33 (11.5%)	17 (2.3%)	50 (4.9%)
Student	137 (47.9%)	300 (41.2%)	437 (43.1%)
Unemployed	13 (4.5%)	67 (9.2%)	80 (7.9%)
Nationality			
Saudi	282 (98.6%)	687 (94.2%)	969 (95.5%)
Not Saudi	4 (1.4%)	42 (5.8%)	46 (4.5%)
Level of education			
University and higher	209 (73.1%)	589 (80.8%)	798 (78.6%)
Secondary	70 (24.5%)	120 (16.5%)	190 (18.7%)
Intermediate	4 (1.4%)	10 (1.4%)	14 (1.4%)
Primary	3 (1.0%)	8 (1.1%)	11 (1.1%)
Not educated	0 (0%)	2 (0.3%)	2 (0.2%)
Family history of FMS	2 (3.0%)	12 (4.6%)	14 (4.3%)
Chronic use of pain reliever	31 (47.0%)	140 (53.6%)	171 (52.3%)
Obesity	21 (31.8%)	58 (22.2%)	79 (24.2%)
Current smoker	16 (24.2%)	8 (3.1%)	24 (7.3%)
Chronic co-morbidities	25 (37.9%)	65 (24.9%)	90 (27.5%)
Average hours of sleep			
Less than 4 hours	6 (9.1%)	27 (10.3%)	33 (10.1%)
4 – 7 hours	46 (69.7%)	174 (66.7%)	220 (67.3%)
8- 10 hours	12 (18.2%)	54 (20.7%)	66 (20.2%)
More than 10 hours	2 (3.0%)	5 (1.9%)	7 (2.1%)
Suffering from psychological pressure because of the chronic pain	12 (18.2%)	52 (19.9%)	64 (19.6%)

**Figure 1:**
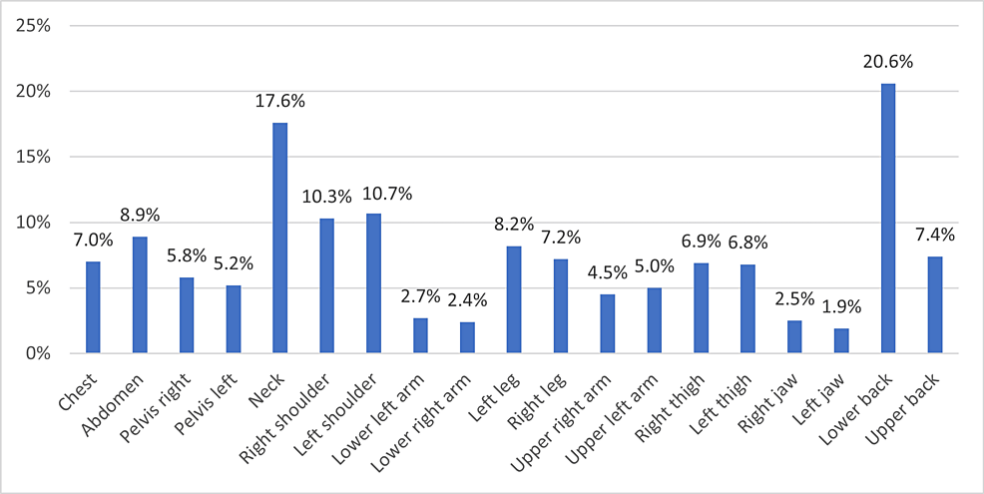
Site of pain felt during the past seven days

A total of 327 participants mentioned that they feel pain in several parts of their bodies for at least three months that is not resulting from an accident or injury. Out of those participants, 52.3% reported chronic use of pain relievers, 19.6% suffer from psychological pressure because of chronic pain, 27.5% suffer from chronic comorbidities, 24.2% are obese, 7.3% are current smokers, and 4.3% mentioned that they have a family history of FMS. More details are provided in Table 1. When asked about the site of pain, 20.6% mentioned lower back, 17.6% mentioned neck, 10.7% mentioned left shoulder, 10.3% mentioned right shoulder, and 8.9% mentioned that they feel pain in their abdomen. More sites of pain are shown in Figure 1.

**Figure 2:**
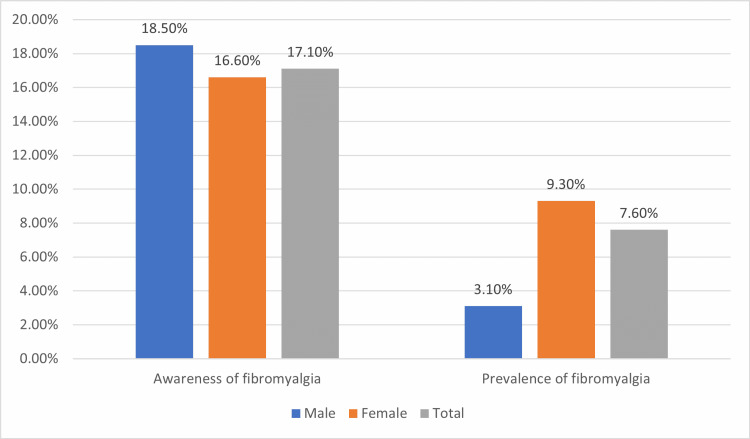
Awareness and prevalence of FMS among the study population FMS: Fibromyalgia Syndrome

The awareness level about FMS was assessed where 17.1% of the participants mentioned that they are aware of FMS or have heard about it before with no significant difference between males (18.5%) and females (16.6%), p=0.462. More details are shown in Figure 2.

Out of 1015 participants, 77 participants (7.6%, 95% CI=5.6%-9.2%) were revealed to have FMS. The prevalence of FMS among females (9.3%) was significantly higher (p<0.001) than that among males (3.1%). In addition, participants aged 40 years old or more showed a significantly higher prevalence of FMS (p=0.003) compared to those aged less than 40 years old (11.7% versus 6.0%, respectively). In addition, occupational status was found to significantly affect FMS prevalence (p=0.040) as the highest prevalence was reported among employees (10.8%) and housewives (9.4%) compared to the unemployed (8.8%), students (5.0%), and retired participants (4.0%). On the other hand, participants’ nationality was shown to have no significant effect on FMS prevalence (p=0.396). More details are provided in Table 2.

**Table 2:** Factors affecting the prevalence of FMS (n=1015); FMS: Fibromyalgia Syndrome

Factors		Have FMS		Do not have FMS		P value
		Count	%	Count	%	
Gender	Male (n=286)	9	3.1	277	96.9	<0.001
	Female (n=729)	68	9.3	661	90.7	
Age group	Less than 40 years (n=732)	44	6.0	688	94.0	0.003
	40 years or more (n=283)	33	11.7	250	88.3	
Nationality	Saudi (n=969)	75	7.7	894	92.3	0.396
	Non-Saudi (n=46)	2	4.3	44	95.7	
Occupation	Employee (n=278)	30	10.8	248	89.2	0.040
	Unemployed (n=80)	7	8.8	73	91.3	
	Housewife (n=170)	16	9.4	154	90.6	
	Retired (n=50)	2	4.0	48	96.0	
	Student (n=437)	22	5.0	415	95.0	

## Discussion

FMS is a common reason for chronic pain. In women, it is considered to be the biggest reason for generalized musculoskeletal pain [22,23]. It is associated with a substantial reduction in health-related quality of life (HRQOL) and disability [24].

The prevalence of FMS in Saudi Arabia is unknown. This cross-sectional study is the first population-based study to investigate the awareness and prevalence rate of FMS in Saudi Arabia. These data are essential as FMS prevalence verifies based on population; in addition, previous observations about FMS were retrieved from the clinics [25,26]. In 2015, the data extracted from "the National Health Interview Survey" had high rates of self-reported pain, comorbidities, and psychological distress [27]. As seen in our study, more than half of the participants revealed chronic usage of pain relievers, and one-fifth reported suffering from psychological pressure because of the chronic pain. Also, almost one-third of individuals mentioned suffering from chronic comorbidities.

Our results concerning the site of pain, the most reported sites were the lower back (20.6%), neck (17.6%), left shoulder (10.7%), right shoulder (10.3%), and finally, 8.9% reported pain in their abdomen. These results were in accordance with another study conducted among working women and housewives [28]. The majority of individuals (42.9%) had pain in muscles, bones, or joints; followed by having pain in shoulders, arms, or hands; then (15.7%) have pain in legs or feet and the least reported was complaining about the neck, chest, or back (4.3%).

There was a lack of awareness about FMS among the Saudi population. The awareness level was estimated to be 17.1%. In addition, no significant difference was shown between males (18.5%) and females (16.6%). Only awareness-level trials performed in Saudi Arabia were conducted among medical practitioners to investigate their ability to diagnose and manage FMS [29,30]. Also in other countries, the assessment of awareness and perception of FMS was set out among rheumatologists or among FMS Patients to explore the relationship between knowledge of HRQOL [31,32]. Data from our study revealed a higher rate of FMS among the Saudi Arabian population (7.6%). Its prevalence in different general populations has been stated to be between 2 and 9% [33].

Our rate is a little higher than most previous western countries. In the United States, the prevalence is approximately 2 to 3 percent [27,34,35]. In Canada, the prevalence of FMS is 3.3%, 1.9% in Germany, 1.30% in Sweden, 0.75% in Finland, and 0.7% in Denmark [33-35]. The higher rates were determined in Tunisia (8.27% and 12.3%) [36].

FMS is commonly more prevalent among women [27]. In our study, FMS was three times more frequent in females than males (9.3% versus 3.1%, respectively (p<0.001). Our results were comparable to previously reported results [36].

However, the rate in women was more prevalent than the previous one (9.3%). In a systematic review and meta-analysis in 2017 of "Prevalence of fibromyalgia in general population and patients", it was found that the total prevalence in women was estimated to be 3.98% [8]. Similarly, in another study, the prevalence in women was between 2.4% and 6.8% [2]. This could be due to the higher rate of FMS in our population (7.6%) in relation to the latter literature (1.8% and 0.2-6.6%, respectively).

Furthermore, FMS commonly increases with age [37]. It is reported that FMS occurs six times more in women than in men aged above 50 [28]. In the current study, a higher significant rate was seen in the age above 40 (11.7% versus 6.0%, respectively). According to the present study, FMS is more likely to be found among employees and housewives than among students, retired and unemployed people (p=0.040) as higher physical and psychological stress is seen while performing tasks. In 2020, a study performed among working women and housewives showed that occupational status has a significant effect on FMS prevalence [28]. Among positive FMS women, 33.3% were working women.

Moreover, our study results further exhibited that the participants’ nationality had no significant effect on FMS prevalence (p=0.396). As per, the National Health Interview Survey, FMS was similar across ethnicities, with lesser rates in Asians [27]. And this result was supported by other reports [2]. One of the limitations of this study was recall bias because our survey questions depend on the history. Also, because there are a lot of residents in Taif city who cannot read or speak Arabic well, so this study did not include them despite they met our inclusion criteria.

## Conclusions

Our results showed a slightly high prevalence rate of FMS in Taif City, Saudi Arabia. Prevalence is seen greater in women, old age, and employed individuals. Poor knowledge of FMS is seen among the general Saudi population. Educational programs are needed to increase awareness of the disease. Also, we recommend further studies to estimate the prevalence of FMS all over the country of Saudi Arabia. 

## Appendices

**Table 3:** QUESTIONNAIRE OF THE STUDY

- Socio-Demographic Data:										
Gender		Male					Female			
Age		20 to 29 years, 30 to 39 years, 40 to 49 years, 50 to 59 years, 60 to 69 years, 70 years and above.								
Nationality		Saudi					Non-Saudi			
Monthly income		Less than 4000 SR, Between 4000 SR to 7000 SR, Between 8000 to 15000 SR, above 15000 SR								
Marital status		Single		Married			Divorced		Widowed	
Educational Level		Primary, Middle, High, Batcheler and above, Non-educated, Others.								
Occupation		Employed, non-employed, Retired, Student, Housewife								
If employed, Type of work		Military, Industrial field, Medical field, Educational field, Others								
- General questions about FMS:										
Are you aware about FM syndrome or heard about it?		Yes					No			
Do you feel pain over most of your body for at least for 3 months?		Yes					No			
If yes answer the followings:										
Has the pain caused by accident or injury ?		Yes					No			
Have you used a medication for this pain ?		Yes					No			
Does this pain affect your exercise ?		Yes					No			
Height:										
Weight:										
Do you have a family history of FM?		Yes					No			
Are you smoker?		Yes					No			
Do you suffer from one of the following chronic diseases - Note: you can choose more than one disease.		HTN, Heart disease, Asthma, Diabetes, I don’t suffer, Others								
Do you suffer from psychological stress from one or more of your family members or friends?		Yes					No			
Have you already diagnosed with FMS?		Yes					No			
- The 2010 American College of Rheumatology Diagnostic Criteria:		0, No problem	1, Slight or mild problems; generally			2, Moderate; considerable problems; often present and/ or at a moderate level				3, Sever: continuous life disturbing problems
During the past week, indicate the severity of the following symptoms		0	1			2				3
Fatigue		0	1			2				3
Trouble thinking or remembering		0	1			2				3
Waking up tired (unrefreshed)		0	1			2				3
During the last six months have had any of the following symptoms?										
Pain or cramps in lower abdomen		Yes						No		
Depression		Yes						No		
Headache		Yes						No		
Please indicate if you have had pain or tenderness over the past seven days in each of the areas listed below, please make a x in the box if you have had pain or tenderness. Be sure to mark both right side and left side separately										
Shoulder-left	Upper leg - left				Lower back					
Shoulder - right	Upper leg - right				Upper back					
Hip - left	Lower leg - left				Neck					
Hip - right	Lower leg - right									
Upper arm - left	Jaw - left									
Upper arm - right	Jaw - right									
Lower arm - left	Chest									
Lower arm - right	Abdomen									
